# Nanostructured Materials for Energy Storage and Conversion

**DOI:** 10.3390/nano12091583

**Published:** 2022-05-07

**Authors:** Luca Pasquini

**Affiliations:** Department of Physics and Astronomy, University of Bologna, Viale C. Berti-Pichat 6/2, 40127 Bologna, BO, Italy; luca.pasquini@unibo.it

The conversion and storage of renewable energy sources is an urgent challenge that we need to tackle to transition from a fossil fuel-based economy to a low-carbon society. I can hardly imagine that this revolution could take place without further breakthroughs in materials science and technology. Indeed, contemporary materials history highlights many game-changing materials that have deeply impacted our lives and contributed to a reduction in carbon dioxide emissions. High-efficiency photovoltaic cells, blue light-emitting diodes, and cathodes for Li-ion batteries are among the most illuminating examples of knowledge-based materials’ development, which have experienced an exponential market permeation and received the highest scientific awards.

These success stories, like many others in materials science, were built upon a tailored control of the interconnected processes that take place at the nanoscale, such as charge excitation, charge transport and recombination, ionic diffusion, intercalation, and the interfacial transfer of matter and charge. Nanostructured materials, thanks to their ultra-small building blocks and the high interface-to volume-ratio, offer a rich toolbox to the scientist that aspires to boost the energy conversion efficiency or the power and energy density of a material. Examples of the materials’ tailoring tools enabled by nanoscience include: (i) the quick separation and collection of photoexcited charges, avoiding recombination issues; (ii) high catalytic activity thanks to the extreme surface area; (iii) accelerated diffusion of ions and atoms along the nanocrystallite interfaces, and (iv) enhanced light harvesting due to the low reflectivity of nanostructured surfaces. Furthermore, new phenomena will arise in nanoparticles (NPs), such as the surface plasmon resonance, which dramatically alters the interaction between metals and the electromagnetic field, superparamagnetism, which turns a ferromagnetic particle into a collective paramagnet, and exciton confinement, which causes the size-dependent colour of semiconductor quantum dots.

The ten articles published in this Special Issue showcase the different applications of nanomaterials in the field of energy storage and conversion, including electrodes for Li-ion batteries (LIBs) and beyond [1,2,3], photovoltaic materials [4,5,6], pyroelectric energy harvesting [7], and (photo)catalytic processes [8,9,10]. The scientific contributions are briefly summarized in the following.

Three main types of anode materials are currently being investigated for the replacement of graphite in LIBs: (i) novel carbonaceous materials, (ii) conversion-type transition metal compounds, and (iii) Si- and Sn-based anodes. Dai et al. report on the electrochemical properties of an ordered array of SnO_2_ nanopillars prepared by pulsed laser deposition on a nanoporous alumina template and employed as conversion-type anode for LIBs [1]. The ordered nanopillar architecture provides adequate room for volumetric expansion during lithiation/delithiation, offering a strategy to mitigate the performance degradation that affects conversion-type anodes. The improved structural integrity and stability allows for a high specific capacity of 524/313 mAh/g to be maintained after 1100/6500 cycles. In the work of Azib et al., the surface chemistry of Si nanoparticles in a Si/Ni_3.4_Sn_4_ composite anode is modified by a coating of either carbon or oxide [2]. The coating strongly reduces the reaction between Si and Ni_3.4_Sn_4_ during the composite’s preparation by ball milling. Better lithiation properties are obtained for carbon-coated Si particles that can deliver over 500 mAh/g for at least 400 cycles. Jahnke at al. studied a highly porous aerogel cathode composed of reduced graphene oxide (rGO), which is loaded with nanostructured SnO_2_ that serves as a cathode material for high-rate Al-ion batteries [3]. This binder-free hybrid has excellent mechanical properties and combines the pseudocapacity of rGO with the electrochemical capacity of SnO_2_ nanoplatelets. The proposed design is appealing for future energy storage devices that can accommodate ionic species other than Li^+^.

The formation of nanoscale features with a high aspect ratio and large surface area on Si is of great interest not only for Si-based battery anodes but also for photovoltaic applications, where the ability to reduce surface reflection leads to enhanced solar harvesting. Kafle et al. report on the formation of such anti-reflective nanostructures, known as “black silicon” features, by the plasma-less etching of crystalline Si using F_2_ gas [4]. This approach may provide an industrial etching tool for products that require a high-volume manufacturing platform. In the field of photovoltaics, Saber et al. present a new approach to the synthesis of nanopowders and thin films of a CuInGaSe_2_ (CIGS) chalcopyrite material doped with different amounts of Cr [5]. From electrochemical impedance spectroscopy measurements, they conclude that thin films with a CuIn_0.4_Cr_0.2_Ga_0.4_Se_2_ composition are promising materials for solar cell applications. Gil et al. report on the synthesis and properties of a hybrid hole transport layer for perovskite photovoltaics, in which high-mobility CuCrO_2_ nanoparticles are incorporated into conventional PTAA. This approach can boost hole extraction while passivating deep-level traps. Moreover, a stability of about 900 h is achieved under 85 °C, 85% relative humidity and continuous 1 sun illumination, suggesting an effective strategy to improve the durability of perovskite solar cells. Pyroelectric energy harvesting, which exploits the temperature changes induced by industrial activity or occurring naturally, can also contribute to the clean energy transition. The work by Krsmanović Whiffen et al. describes a co-precipitation method for the synthesis of nanocrystalline wurtzite ZnS, which is an interesting material for pyroelectric applications [7].

Three papers in this Special Issue focus on thermocatalysis, photocatalysis, and plasmon-driven catalysis. Calizzi et al. studied the thermocatalytic hydrogenation of CO_2_ by Fe-Co alloy nanoparticles with different compositions, as prepared by inert gas condensation [8]. The Fe-Co nanoalloys can catalyse the formation of C_2_-C_5_ hydrocarbons, which are not detected using elemental Co and Fe nanoparticles. This effect is attributed to the simultaneous variations in CO_2_ binding energy and decomposition barrier as a function of the Fe/Co ratio in the nanoalloy. Mazzanti et al. report on the reductive cleavage of azo bonds by the UV photoexcitation of nanostructured TiO_2_ films in contact with an aqueous solution of azo dyes [9]. Charge separation is extremely long-lived in nanostructured TiO_2_ thin films, making them suitable for driving both oxidation and reduction reactions. This approach provides an effective solution for the simultaneous implementation of wastewater purification and photocatalytic conversion of waste into useful products. Finally, plasmonic nanoparticles have recently attracted interest in the field of photocatalysis thanks to their ability to harvest and convert light into highly energetic charge carriers and heat. The perspective article written by Hamans et al. highlights two techniques for single-particle studies of structure–function relations between plasmonic nanocatalysts: surface-enhanced Raman spectroscopy and super-resolution fluorescence microscopy [10]. These two far-field optical techniques make it possible to take a closer look at fundamental nanoscale processes, such as photoactivation mechanism, molecular intermediates, and reaction pathways.

I wish to express my deepest gratitude to all the authors who contributed to this Special Issue and to the reviewers who generously dedicated their time to improving the quality of the submitted manuscripts. Special thanks go to all the editorial staff of *Nanomaterials* for their enduring support during the preparation and publication of this Special Issue.
